# Supplementary material for: Use of Preliminary Exposure Reduction Practices or Laundering to Mitigate Polycyclic Aromatic Hydrocarbon Contamination on Firefighter Personal Protective Equipment Ensembles
Source: Int J Environ Res Public Health. 2023 Jan 24;20(3):2108. doi: 10.3390/ijerph20032108 (PMC9916157; doi:10.3390/ijerph20032108)

Table S1. Comparison of PAHs (μg/100 cm^2) collected from turnout gear using 70% isopropyl alcohol (IPA) and benzalkonium chloride (BA) wipes after a smoke exposure scoping experiment.

Sampling Site	Type of wipe	Collection period relative to smoke exposure	N	Median	Range
Outer Shell Jacket (sleeve)	BA	After	3	14.02	7.65 – 16.55
	IPA	After	3	8.21	7.61 – 13.17
Outer jacket (chest)	BA	After	3	15.56	12- 15.72
	IPA	After	3	10.44	7.74 – 11.31
Helmet	BA	After	1	56.35	N/A
	IPA	After	1	57.63	N/A

Table S2. Collection efficiency of wipes at measuring PAHs (μg/cm^2) from baking sheet.

Sampling Method	N	Median	Range	% Referent (Median, Range)
PTFE Filter	4	42.78	37.85 – 62.67	Ref
Wipes	4	61.32	34.97 – 108.8	145.2 (89.78 – 180.1)

Table S3. Median levels (μg/100 cm^2) of the most dominant PAHs (Fluorene, Naphthalene, Phenanthrene, and Pyrene) collected from two consecutive wipes of firefighter gear after repeated exposures and cleanings[*].

Sampling Site	Treatment	Timing	N	N of Non-Detects	Median	Range	P-Value[†]
Exterior Jacket Outer Shell (N=21)	Laundered	Before	3	2	<LOD	<LOD – 1.00	Reference
		After	6	0	2.69	2.50 – 5.72	<.001
	Wet Soap PER	Before	3	0	1.66	0.90 – 1.73	Reference
		After	9	0	2.90	0.42 – 6.25	0.188
Jacket (N=19)	Laundered	Before	4	4	<LOD	<LOD	Reference
		After	8	7	<LOD	<LOD – 0.27	Not Applicable
	Wet Soap PER	Before	3	2	<LOD	<LOD – 0.29	Reference

	Decontaminated	After	4	1	0.27	<LOD – 0.33	Not Applicable
Pant Outer Shell (N=11)	Laundered	Before	3	2	<LOD	<LOD – 0.27	Reference
		After	3	1	0.51	<LOD – 1.38	0.142
	Wet Soap PER	Before	3	3	<LOD	<LOD	Reference
		After	2	1	0.85	<LOD – 1.70	0.071
Helmet (N=13)	Wet Soap PER	Before	6	1	1.18	<LOD – 3.81	Reference
		After	7	0	12.17	3.77 – 25.20	<.001

† Non-detectable concentrations below the limits of detection (LOD) were assigned values using the β-substitution method (Ganser and Hewett, 2010). Maximum likelihood estimation method (Helsel, 2006) via SAS procedure LIFEREG was utilized to determine if the total PAH concentrations between before vs. after by sample site and treatment were significantly different. P-value was specified as "Not Applicable" when the analysis modeling was not convergent.

Supplemental Figures

Figure S1. PTFE filter on baking sheet.

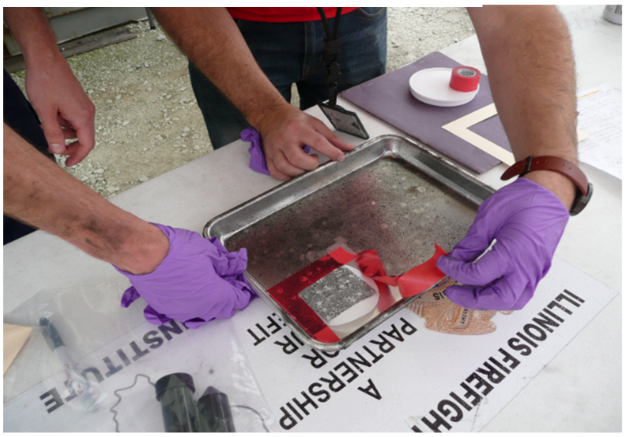

Figure S2. Baking sheet wipe area and template.

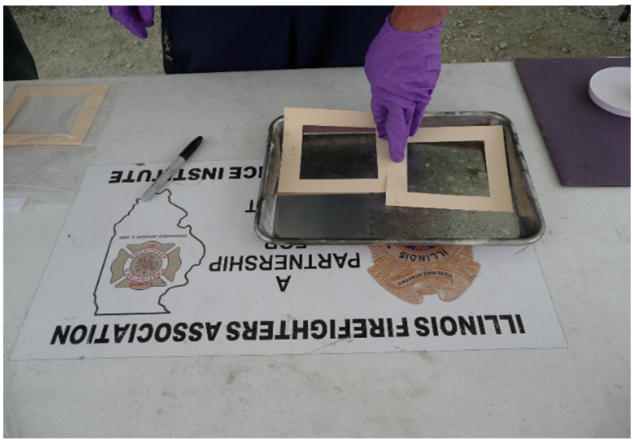

Figure S3. Side-by-side comparison of zipper and hook & dee turnout jacket

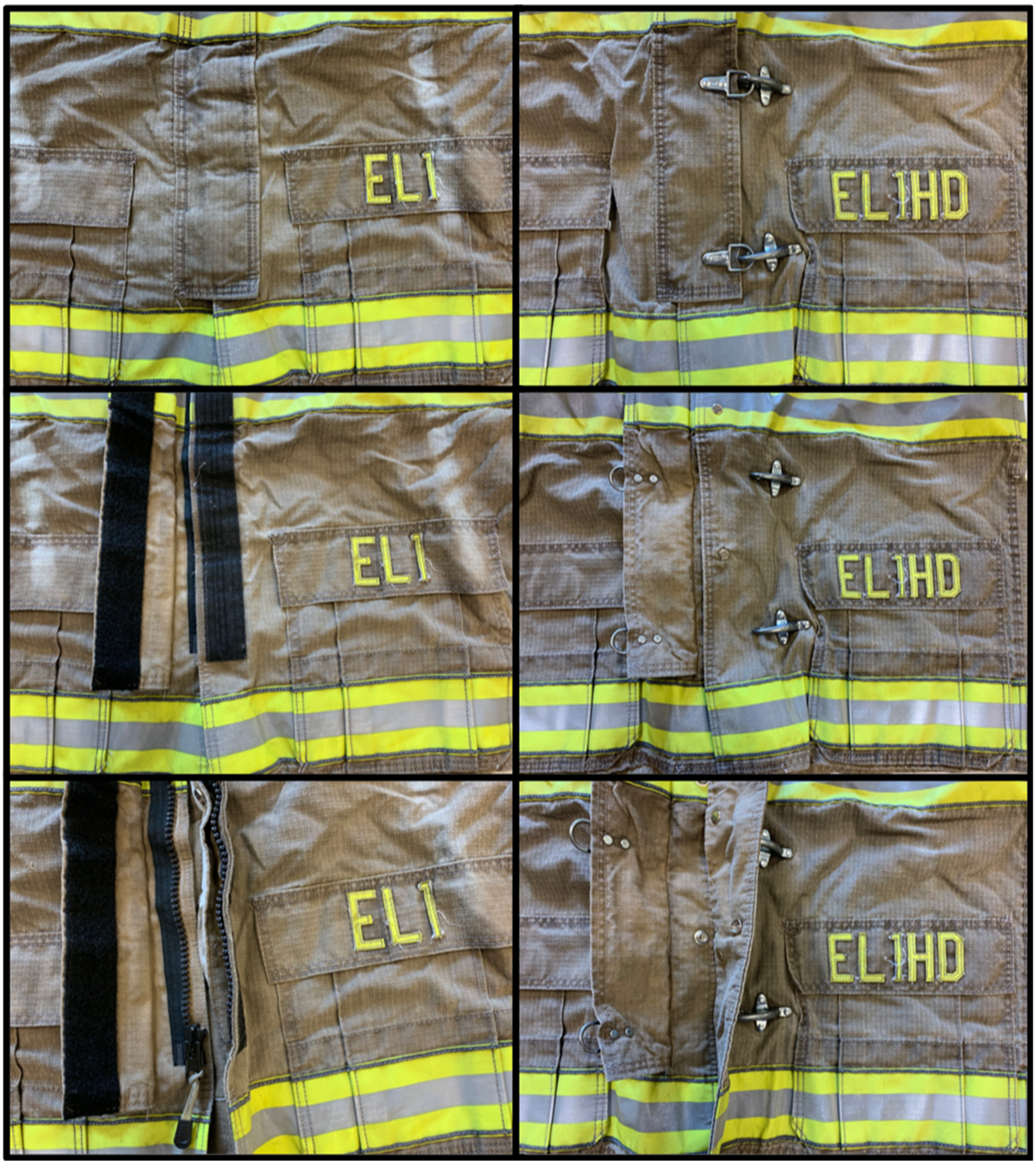

Supplement: Supplementary file 1 [file ijerph-20-02108-s001.zip › ijerph-2132611-supplementary.pdf]
